# Emerging Potential of Exosomes on Adipogenic Differentiation of Mesenchymal Stem Cells

**DOI:** 10.3389/fcell.2021.649552

**Published:** 2021-06-22

**Authors:** Yuxuan Zhong, Xiang Li, Fanglin Wang, Shoushuai Wang, Xiaohong Wang, Xiaohong Tian, Shuling Bai, Di Miao, Jun Fan

**Affiliations:** ^1^Department of Tissue Engineering, School of Fundamental Science, China Medical University, Shenyang, China; ^2^Department of Cell Biology, Key Laboratory of Cell Biology, Ministry of Public Health, Key Laboratory of Medical Cell Biology, Ministry of Education, China Medical University, Shenyang, China; ^3^China Medical University–The Queen’s University of Belfast Joint College–Combination, Shenyang, China

**Keywords:** exosomes, mesenchymal stem cell, adipogenic differentiation, adipogenesis, regenerative medicine

## Abstract

The mesenchymal stem cells have multidirectional differentiation potential and can differentiate into adipocytes, osteoblasts, cartilage tissue, muscle cells and so on. The adipogenic differentiation of mesenchymal stem cells is of great significance for the construction of tissue-engineered fat and the treatment of soft tissue defects. Exosomes are nanoscale vesicles secreted by cells and widely exist in body fluids. They are mainly involved in cell communication processes and transferring cargo contents to recipient cells. In addition, exosomes can also promote tissue and organ regeneration. Recent studies have shown that various exosomes can influence the adipogenic differentiation of stem cells. In this review, the effects of exosomes on stem cell differentiation, especially on adipogenic differentiation, will be discussed, and the mechanisms and conclusions will be drawn. The main purpose of studying the role of these exosomes is to understand more comprehensively the influencing factors existing in the process of stem cell differentiation into adipocytes and provide a new idea in adipose tissue engineering research.

## Introduction

Exosomes are membrane vesicles released to the extracellular environment after fusion of multivesicular endosomes and plasma membranes, and all types of cells can produce exosomes under normal or pathological conditions. The study of exosomes lasted for nearly 40 years. Trams et al. (1981) found exfoliated vesicles from cultured normal cells and tumor cells with an average diameter of 500–1000 nm, and suggested that these vesicles with physiological functions be called “exosomes.” Johnstone et al. (1987) named them “exosomes.” Due to the shortage of technology and insufficient understanding of exosomes, most of the exosomes studied in early years came from mammalian erythrocytes (Bette-Bobillo and Vidal, 1995) or lymphocytes (Raposo et al., 1996). With the deepening understanding of the composition and mechanism of exosomes, various cell-derived exosomes have triggered relevant studies. In general, exosomes are widely used as a kind of nanocarrier to treat tumors by delivering nucleic acids or drugs (Lim et al., 1997; Wang D. et al., 2020). However, with the development of cosmetic and regenerative medicine, there are also studies showing that exosomes secreted by stem cells can promote tissue and organ regeneration. Therefore, more and more researchers are involved in the study of exosomes roles in tissue regeneration (Zhang et al., 2017).

Mesenchymal stem cells (MSCs) are pluripotent adult stem cells with the potential of multilineage differentiation. They can differentiate into adipose, bone, muscle, nerve, and endothelial tissue cells under specific induction conditions (Caplan, 1991). MSCs are rich sources and have strong proliferation ability, and immune regulatory function. Therefore, the application of MSCs in regenerative medicine research is endless.

Adipogenic differentiation is an important direction of MSCs differentiation which has attracted much attention in recent years. Adipogenic differentiation plays an important role in the regeneration of damaged organs and the repair of adipose tissue (Zhao et al., 2017; Morissette Martin et al., 2018; Suzuki et al., 2019). Studies demonstrate that microRNAs and glucocorticoids are related with adipogenic differentiation (Han et al., 2019; Lorente-Cebrian et al., 2019). In addition, recent studies indicate that exosomes also play a significant role in regulating the adipogenic differentiation of MSCs. Exosomes promote or inhibit adipose production by delivering genes or proteins associated with fat formation (Zhang et al., 2017; Zuo et al., 2019).

In this review, we discussed the effect of exosomes on the adipogenic differentiation of MSCs based on the existing research, combined with the function of exosomes and the mechanism of adipogenic differentiation of MSCs, and summarized the mechanism and pathway of the influence, providing a reference for the study of exosomes effect on adipogenic differentiation of MSCs.

## Mechanisms of Adipogenic Differentiation of Mesenchymal Stem Cells

Adipogenesis is a complex process involving many transcription factors and regulatory genes (Moseti et al., 2016). During the process of adipogenic differentiation, the morphology and function of mesenchymal stem cells change to adipocytes and cannot differentiate into osteocytes, myocytes and other cells (Chen et al., 2016; Nic-Can et al., 2019). Adipogenesis is mainly divided into two stages: commitment and terminal differentiation. MSCs formed pre-adipocytes and then eventually differentiate into mature adipocytes (Ambele et al., 2020; Wang F. et al., 2020). Specific detailed differentiation steps can be referred to the summary of our laboratory (Wang F. et al., 2020).

## Characteristics and Functions of Exosomes

Exosomes are widely present in body fluids. Exosomes are one type of extracellular vesicles (EVs), which are nanoscale vesicles with diameters between 30 and 150 nm. The release process of exosomes was first discovered during the differentiation of reticulocytes, and then it was found that B lymphocytes and dendritic cells could also produce exosomes (Harding et al., 1983; Raposo et al., 1996; Liang et al., 2019). The release of exosomes requires three stages: the plasma membrane invaginates to form early endosomes, then forms multivesicular bodies (MVBs) under the action of endosomal sorting complex (ESCRT), and finally fuses MVB with the plasma membrane to release exosomes (Hessvik and Llorente, 2018). The contents of exosomes include proteins, lipids, RNA and DNA (Lin et al., 2015).

### Isolation and Characterization of Exosomes

The separation of exosomes from body fluids and the medium is an essential step, for the size of exosomes is too small to isolate. It is a very challenging task. There are many methods to separate exosomes, and the most suitable method can be selected according to the experimental needs. Ultracentrifugation is the most widely used separation method. It is easy to operate, inexpensive and time-consuming. Size-based methods for separating exosomes include ultrafiltration (UF), polymer precipitation, and size-exclusion chromatography (SEC), which only depends on size or molecular weight. UF is often used for size-based isolation of exosomes, which is time-saving and produces high purity exosomes. To compensate for the UF’s difficulty in removing contaminated proteins, it is often used in combination with the ultracentrifugation (Kim et al., 2020). In addition, some studies have shown that a combination of ultrafiltration and size exclusion chromatography can greatly increase exosome production (Oeyen et al., 2018; Shu et al., 2020). Immunoaffinity purification requires fewer samples, is rapid, easy, and compatible with conventional laboratory equipment (Nakai et al., 2016). In addition to these conventional methods, it is important to develop new techniques to provide high purity exosomes. Microfluidics-based isolation technology utilizes innovative sorting mechanisms such as acoustic, electrophoretic, and electromagnetic manipulations to significantly reduce sample quantity, time consumption, and experimental reagents (Dorayappan et al., 2019). These methods are summarized and compared as follows (Table 1).

**TABLE 1:** Comparison of isolation methods of exosomes.

	Isolation principle	Advantages	Disadvantages	References
Ultracentrifugation	Separate particle composition according to density, size, and shape	It is suitable for large sample volumes and yields large amounts of exosomes.	Not suitable for the small volumes of clinical samples. Labor intensive and low portability.	Lobb et al., 2015; Li et al., 2018
Ultrafiltration (UF)	Based solely on size differences between exosomes and other particulate components	Short time consuming and high purity, does not require expensive equipment.	Difficult to remove contaminated proteins.	Oeyen et al., 2018; Kim et al., 2020
Size exclusion method (SEC)		High purity and retaining biological activity.	Requires dedicated equipment. Not suitable for the enrichment of exosomes.	Koh et al., 2018; Monguio-Tortajada et al., 2019
Precipitate		Suitable for large sample size, easy to operate without special equipment	There is no specificity for non-exosomal material and low purity.	Soares Martins et al., 2018; Coughlan et al., 2020
Immunoaffinity purification	Exosomes are isolated using interactions between proteins (antigens) and their antibodies, and specific interactions between receptors and ligands.	Rapid, easy, and have greater separation efficiency and increased sensitivity.	High cost, low yields. Need for exosome tags.	Greening et al., 2015; Zarovni et al., 2015; Nakai et al., 2016
Microfluidic-based isolation technology	Common isolation determinants, such as size, density, and immunoaffinity but also innovative sorting mechanisms, such as acetic are exploited	Fast, low cost and easy automation	Requiring some expertise. It is obstructed by standardization.	Contreras-Naranjo et al., 2017; Dorayappan et al., 2019
				

**FIGURE 1:**
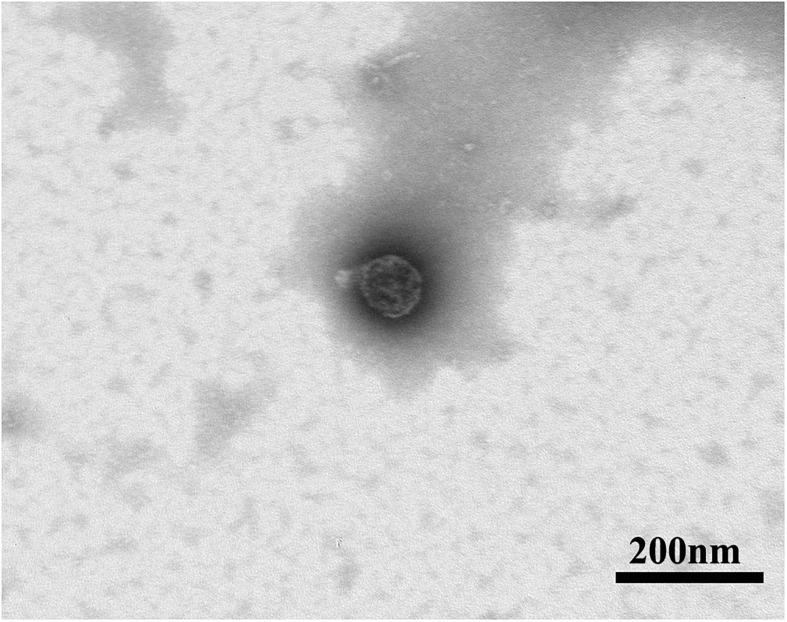
Representative micrographs of transmission electron microscopy obtained from purified rat MSCs-derived exosomes (scale bar: 200 nm).

Several methods are used for the detection of yield and purity of exosomes such as protein staining, Western blot, and proteomic techniques (Gheinani et al., 2018). In order to observe and evaluate the morphological structure and chemical characteristics of exosomes, different techniques have been used to characterize exosomes, such as dynamic light scattering (DLS) and nanoparticle tracking analysis (NTA) for characterizing their size, atomic force microscopy (AFM) and transmission electron microscopy (TEM) for observing the morphology and biomolecular components of exosomes (Figure 1), and flow cytometry for characterizing protein molecules on the surface of exosomes (Jung and Mun, 2018; Nolan and Duggan, 2018; Skliar and Chernyshev, 2019). Raman spectroscopy was used to characterize the chemical structure of exosomes (Carmicheal et al., 2019). Flow cytometry is the most commonly used method to analyze the biological characteristics of vesicles. These methods are summarized and compared as follows (Table 2).

**TABLE 2:** Comparison of characterization methods of exosomes.

	Purpose of characterization	Advantages	Disadvantages	References
Western blot	For the detection of yield and purity of exosomes	It is easy for use, and have the ability to detect exosomal surface proteins and internal proteins	It’s impossible to observe the intact vesicles	Szatanek et al., 2017
DLS	For estimating particle size and concentration of exosomes	It is able to measure particles ranging in size from 1 nm to 6 μm	Analysis of heterogenous mixtures is not easily performed	Sokolova et al., 2011
NTA	For estimating particle size and concentration of exosomes	very quick and easy using	The original form could not be determined	Dragovic et al., 2011
AFM/TEM	For observing the morphology and biomolecular components of exosomes	The demand of sample preparation is small and the operation is not destructive	Different samples require different experimental conditions, such as temperature, force between probe and sample	Yuana et al., 2010; Wu et al., 2015
Flow cytometry	For characterizing exosomal surface proteins and measuring the size and structure of exosomes	For analyzing the physical characteristics and chemical characteristics of exosomes	It has limitations in terms of size, small diameter particles cannot be detected	Groot Kormelink et al., 2016

### Function of Exosomes

Exosomes are released from a variety of cells and exist widely in the body fluids of humans and other organisms. Their biological function and specificity depend on the type and state of the cells that release them. Because exosomes can transfer RNA, proteins, and lipids, they have many biological functions. Exosomes can affect cell proliferation, differentiation, apoptosis and migration. It was shown that MSC-derived exosomes promoted the growth of skin cells by increasing the phosphorylation of extracellular signal-regulated kinase (ERK)-1/2 (Kim et al., 2018). MSC exosome also promote proliferation and reduce apoptosis of adjacent cells in repairing cartilage loss (Zhang S. et al., 2018). In further, exosomes from cancer cells can induce cell migration of liver cancer cells (Chiba et al., 2016). There are also studies showing that exosomes also play a role in the immune response and inflammatory response Lv et al. (2018). For example, adipose tissue-derived stem cells (ADSCs)-derived exosomes promote macrophage polarization toward M2 phenotypes (Zhao et al., 2018). The exosomes produced by human embryonic stem cell derived MSCs can ameliorate interleukin-1beta (IL-1-β)–induced inflammatory effects (Zhang S. et al., 2019). In addition, exosomes also play an important role in angiogenesis. Exosomes released from chronic myelogenous leukemia cells regulate the process of neovascularization by activating the mitogen-activated protein kinase (MAPK) signaling pathway (Taverna et al., 2012). Exosomes from colorectal cancer cells contained miR-25-3p, which regulate vascular permeability and angiogenesis by suppressing the expression of Kruppel-like factor 2 (KLF2) and Kruppel-like factor 4 (KLF4) (Zeng et al., 2018). The sources and functions of several common exosomes are summarized as following (Table 3).

**TABLE 3:** The sources and functions of exosomes.

Function	Sources	Related Genes and Proteins	Applications	References
Influence cell proliferation and differentiation	Stem cell	miR126	Increasing the production of hematopoietic stem cells (HSCs) for clinical use	Liao et al., 2018
		The PI3K/Akt signaling pathway	Promote wound healing	Zhang W. et al., 2018
		microRNA profiles	Treatment of bone tissue defects	Wang et al., 2018b
Destroy cell integrity and inhibit cell apoptosis	Human embryonic stem cell-derived MSCs	–	Promote cartilage repair and regeneration	Zhang S. et al., 2018
	Cardiac-resident progenitor cells (CPCs)	Pregnancy-associated plasma protein-A (PAPP-A)	Reduce cardiomyocyte apoptosis and protect the heart	Barile et al., 2018
Mediates cell migration	Colorectal cancer cells	MAP	It is helpful to explore the interaction between cancer cells and adjacent normal cells *in vivo*	Chiba et al., 2016
As a biomarker	Colorectal cancer (CRC) cells	CK19	As special markers for CRC	Xiao et al., 2019
	Placental Exosomes	hsa-miR-486-1-5p, hsa-miR-486-2-5p	Prevention of preeclampsia in pregnant women	Salomon et al., 2017
Presentation of antigens	Dendritic cells		Increased or decreased immune response with medical treatment	Shenoda and Ajit, 2016; Lindenbergh and Stoorvogel, 2018
	Human embryonic kidney cells 293 (HEK293)	Lactadherin (LA), group-specific antigen (Gag)	The loading of antigen proteins inside exosomes helps in efficient antigen presentation	Arima et al., 2019
Mediate endocytosis	Macrophage	GFP, T cell immunoglobulin and mucin receptor 1	Transfer of content by fusion of exosomes with the plasma membrane, as an effective carrier of antiviral therapy	Yao et al., 2018
Drug loading	Bone marrow mesenchymal stem cells (BM-MSC)	miR-125b-5p	As a novel drug carrier that enhances the specificity of drug delivery	Zhu et al., 2018
Involvement in inflammatory responses	Human embryonic stem cell	IL-1 β	Relief of temporomandibular joint osteoarthritis	Zhang S. et al., 2019
Regulation of angiogenesis	Chronic myelogenous leukemia	MAPK	Exosomes induce endothelial cell ingrowth and vascularization of Matrigel plugs in mice	Taverna et al., 2012
	Colorectal cancer cells	miR-25-3p	Promotes vascular permeability and angiogenesis	Zeng et al., 2018

## The Effect of Exosomes on the Differentiation of MSCs

The exosomes can specifically bind to target cells and regulate stem cell differentiation by increasing intercellular communication. Transcription factors, growth factors, and certain specific miRNAs and proteins contained in exosomes will affect differentiation of stem cells (Reilly and Engler, 2010; Whiteside, 2018; Chew et al., 2019). The exosomes can achieve stem cell lineage-specific differentiation. Narayanan et al. (2018) demonstrate that osteoblast-derived exosomes can accelerate osteogenic differentiation and increase the expression of osteogenic genes several times, but adipocyte-derived exosomes do not affect the expression of osteogenic genes. In a recent study, Zhu W. et al. (2020) found that exosomes extracted from non-traumatic osteonecrosis of the femoral head (ONFH) tissues inhibited osteogenic differentiation of murine MSCs due to lack of integrin CD41. In addition, exosomes secreted by human cholangiocarcinoma cells can induce fibroblastic differentiation in human bone marrow MSCs (h BM-MSCs) (Haga et al., 2015).

### Properties of Exosomes Derived From MSCs

The exosomes secreted by MSCs first triggered research in 2010, which could reduce myocardial ischemia injury in mice, and then triggered many related studies, such as adipose stem cells secreted exosomes promoted angiogenesis (Lai et al., 2010; Zhang J. et al., 2018; Xu et al., 2019). There was no difference in morphology and isolation method between MSCs and other exosomes. MSCs secreted much more exosomes than other cells in terms of yield (Yeo et al., 2013). Besides, its surface markers include not only the CD9 and CD81 expressed by exosomes, but also some adhesion molecules, such as CD29, CD44, and CD73, expressed on the MSCs membrane (Lai et al., 2012). MSCs protein components in derived exosomes do not remain constant and aggregate according to the function of these proteins (Qiu et al., 2019). MiRNA in MSCs-derived exosomes mainly exist in their precursor form, and these miRNAs can participate in the biological activity of certain cells. The miR-143 contained in human MSCs secreted exosomes can be transferred to prostate cancer cells and inhibit prostate cancer by promoting its apoptosis. MiR-182 contained in mesenchymal stem cell-derived exosomes can alter macrophage polarization (Che et al., 2019; Zhao et al., 2019).

### Ways of Exosomes Involvement in MSCs Differentiation

#### Endocytosis of Exosomes

The exosomes can gain entry into target cells by way of fusion and/or endocytosis. The primary internalized mechanism is endocytosis, which includes macropinocytosis, clathrin-dependent endocytosis and clathrin-independent pathways (Gonda et al., 2019). MSCs can affect some specific protein and RNA levels in cell differentiation by endocytosis of exosomes (Svensson et al., 2013). It has been shown that fibroblasts can endocytose umbilical cord-derived exosomes and then inhibit myofibroblast formation by blocking the transforming growth factor-β2/SMAD2 pathway (Fang et al., 2016). But the specific way of its endocytosis has not been explored. Macropinocytosis of exosomes is dependent on Na^+^ and phosphatidylinositol 3-kinase (PI3K) (Tian et al., 2014). Exosomes size and cell type determine whether to use clathrin or caveolin. Commonly, exosomes smaller than 60 nm are absorbed by the endocytosis of caveolin, while clathrin-dependent mechanisms can internalize exosomes up to 120 nm. Exosomes have tissue properties, such as hepatocytes rarely ingesting exosomes from non-hepatocytes (Yao et al., 2018). In general, exosomes can be used to affect stem cell differentiation by mediating endocytosis. The pathway of endocytosis depends on the cell type, the receptor on the cell surface, and the size of the exosomes.

#### Exosomes Binding to Extracellular Matrix (ECM) Proteins

Extracellular matrix (ECM) is a collagen-based structure containing a variety of proteins that can support cellular activities. ECM can serve as a bridge for cell communication and also influence cell differentiation fate (Rittie, 2015; Walker et al., 2018). Exosomes are invaginated vesicles in the plasma membrane, so the membrane of exosomes also belongs to the plasma membrane. Integrins and other receptors on the plasma membrane surface can bind to ECM proteins to promote MSCs differentiation (Clayton et al., 2004; Lin et al., 2019). It has been reported that exosomes can significantly up-regulate Runx2 and Osterix expression and promote MSCs osteogenic differentiation by binding to type I collagen hydrogel (Narayanan et al., 2016). Moreover, the combination of exosomes and tissue specific ECM switches on the lineage gene expressions at earlier time points of differentiation process compared to the exosomes or ECM conducted individually (Narayanan et al., 2018). Exosome binding to ECM can also promote organ function repair. For example, Zhang et al., found that combinatory transplantation of exosomes derived from gingival mesenchymal stem cells (GMSCs) and small intestinal submucosa-extracellular matrix (SIS-ECM) could promote the restoration of taste bud function (Zhang Y. et al., 2019). So, the exosomes may be used in combination of decellularized biological scaffolds to improve their performance.

## Exosomes Regulate Adipogenic Differentiation

The microenvironment of cell growth includes exosomes secreted by cells and various transforming factors, which are closely related to the differentiation of MSCs (Pittenger et al., 1999; Reilly and Engler, 2010). The proteins and miRNAs contained in exosomes play a decisive role in the various differentiation directions of MSCs (Narayanan et al., 2018). Exosomes contain osteogenesis-related miR-34a, miR-27a, and miR-22, adipogenesis-related miR-143 and miR-375 and other miRNAs (Shi et al., 2016; Narayanan et al., 2018). Therefore, based on the understanding of exosomes, many related studies on osteogenesis and adipogenesis have been initiated (Zhao et al., 2017; Zuo et al., 2019). Recent studies have found that some exosomes participate in adipogenic differentiation by being endocytosed by cells, and some exosomes can regulate adipogenesis by binding to ECM proteins. The mechanisms on exosomes affecting adipogenesis were summarized as follows (Table 4).

**TABLE 4:** Exosomes regulate adipogenic differentiation.

Exosome source	RNA or Protein	Target genes or Signaling pathways	Target cells	Regulatory results	References
Rat adipose tissue	miR-450a-5p	WISP2	ADSCs	↑ Adipogenesis	Zhang et al., 2017
M1 macrophage	–	PPAR- gamma	BM-MSCs	↑ Adipogenesis	Xia et al., 2020
Fetal bovine serum (FBS)	miR-1246	EBF1	BM-MSCs	↓ Adipogenesis	Zhou et al., 2020
Gastric cancer cell (GC)	ciRS-133 miR-133	miR-133/PRDM16	–	↑ Adipogenesis	Zhang H. et al., 2019
Lung tumor A549	–	TGFβ	ADMSCs	↓ Adipogenesis	Wang et al., 2017
Tumor cell line K562	miR-92a-3p	C/EBPα	Mouse ADSCs	↓ Adipogenesis	Wan et al., 2019

### Adipose Tissue Exosomes→miR-450a-5p→WISP2

A recent study demonstrates that exosomes secreted by rat adipose tissue contain abundant miRNAs that regulate cell differentiation. QPCR assays showed that the contents of miR-450a-5p, miR-99a-5p, and miR-30a-5p were the most abundant (Zhang et al., 2017). In particular, miR-450a-5p plays an important role in adipogenesis. Among the targets of miR-450a-5p, WNT1 inducible signaling pathway protein 2 (WISP2) was verified to be involved in adipogenesis (Hammarstedt et al., 2013). MiR-450a-5P can target the 3′UTR of WISP2 to inhibit the expression of WISP2 (Bowers and Lane, 2007). Experiments showed that WISP2 was down-regulated with the up-regulation of miR-450a-5p during adipogenic culture of rat ADSCs. WISP2 is a classical WNT mediator that interacts with BMP4 to inhibit adipogenic differentiation in stereotyped 3T3-L1 pre-adipocytes (Xue et al., 2014). Inhibition of WISP2 by miR-450a-5p induces spontaneous adipogenic differentiation of stem cells.

Adipose tissue-derived stem cells promote adipogenesis only when ingesting adipose tissue-derived exosomes, but not when ingesting ADSCs-derived exosomes (Zhang et al., 2017). Other studies have found that exosomes from ADSCs transfer between macrophages rather than ADSCs (Zhao et al., 2018). Therefore, we can conclude that the transfer of exosomes is unidirectional, only unidirectional transfer from secreted cells to target cells. This suggests that the exosomes will not be absorbed by parental cells when they are used as carriers to deliver the contents to the target cells.

### M1 Macrophage Exosomes→PPAR-Gamma

Macrophages are important participants in the immune response (Mosser and Edwards, 2008). Non-polarized (M0) macrophages can switch to polarized (M1 and M2) macrophages as needed when participating in various responses (Mantovani et al., 2013). Exosomes secreted by macrophages of all three phenotypes can be internalized by BM-MSCs and then participate in cell differentiation (Xia et al., 2020). Macrophages also play important roles in adipogenesis process. Early studies showed that M1 macrophages inhibited adipogenesis in PDGFRα^+^ pre-adipocytes (Cheng et al., 2019). Recent studies have reported that exosomes secreted by M1 macrophages can significantly up-regulate the expression of PPAR-gamma gene to promote lipid droplet formation (Xia et al., 2020). However, exosomes secreted by M0 and M2 macrophages had negative effects on the lipid droplet formation of the BM-MSCs (Xia et al., 2020). In further, previous study demonstrated that the condition medium of M1 macrophage could promote the adipogenic differentiation of BM-MSCs. This suggested that exosomes may be involved in regulating the effect of macrophages-derived medium on the BM-MSCs’ adipogenic differentiation.

According to the above studies, we conclude that the exosomes derived from macrophages can regulate adipogenesis process. This indicates that macrophages-derived exosomes may be as a candidate to promote the adipogenic differentiation of MSCs in the regenerative microenvironment.

### FBS Exosomes→miR-1246

Fetal bovine serum (FBS) is the common additives in the cell culture medium. FBS contains high levels of growth factors which provides essential active ingredients for cell growth (Samareh Salavati Pour et al., 2020). Various kinds of regulatory RNAs including mRNA, miRNA, rRNA and so on were found in FBS (Wei et al., 2016). There is also a large number of EV in the FBS supplements, which contains miRNA, such as miR-122, miR-451a, and miR-1246 (Lehrich et al., 2018). Although high-speed centrifugation and other methods are used to remove PBS EV, some vesicles are still left, such as exosomes (Lehrich et al., 2018). FBS-derived exosomes exist in the microenvironment of cell growth, so its function cannot be ignored. A recent study demonstrated that FBS-derived exosomes could directly inhibit the adipogenic differentiation of the human bone marrow mesenchymal stromal cells (h BM-MSCs), and miR-1246 transferred by FBS exosomes may partly contribute to this effect. MiR-1246 has been shown to be rich in FBS secreted exosomes (Wei et al., 2016). MiR1246 can target early B cytokine 1 (EBF1) by binding to the site of EBF1 3′UTR. Experiments have identified EBF1 as a transcription factor expressed by adipocytes, which can promote fat decomposition (Gao et al., 2014).

These results suggest that FBS exosomes are the negative regulator of adipogenic differentiation. When the medium of FBS additive is used in cell culture, the exosomes secreted by the FBS has a certain effect on the cell culture, and it should be included in the consideration of the influencing factors regulating lipogenesis.

### Gastric Cancer (GC) Exosomes→ciRS-133→miR-133→PRDM16

At present, non-coding RNAs have been explored to be rich in exosomes. In addition to miRNA, a class of non-coding RNA, circular RNA, has also been found to be used to participate in the role of exosomes. Circular RNAs are produced by precursor mRNAs (pre-mRNAs) that have been shown to participate in lipid, osteogenic, and myogenic differentiation in cells (Wang Y. et al., 2019; Chen et al., 2020). Previous studies have shown that circ RNA is enriched and stable in exosomes (Li et al., 2015). A recent study found significant up-regulation of Hsa_circ_0010522 in GC exosomes, which was named ciRS-133 because of its interaction with miR-133 that ciRS-133 is co-localized with miR-133. PR domain containing 16 (PRDM16), a zinc finger transcription factor, has been proposed to promote browning of white adipose tissue (WAT) (Van Nguyen et al., 2020). MiR-133 has been shown to be the upstream regulator of PRDM16, suppressing PRDM16 expression by targeting PRDM16 3′UTR in adipocytes. However, GC exosome-delivered ciRS-133 suppress the functionality of miR-133 to promote PRDM16 expression (Zhang H. et al., 2019). By inhibiting Mir-133 to activate PRDM16, ciRS-133 activates uncoupling protein 1 (UCP1) and promotes pre-adiponectin browning (Yin et al., 2013; Zhang H. et al., 2019).

In addition, circular RNA can be used to modify ADSC exosomes to participate in cell differentiation (Zhu M. et al., 2020). This suggests that circular RNA could be an effective component of exosome lipid regulation.

### Lung Tumor Exosomes→TGFβ

Transforming growth factor β (TGFβ) signaling plays an important role in adipogenic and osteogenic differentiation. Transforming growth factor β (TGFβ1 and TGFβ2) are both responsive genes in the TGF-β pathway (Qian et al., 1987). TGFβ1 prevents adipogenic differentiation by inhibiting the expression of clade B (ovalbumin) member 2 (SERPINB2) which can promote cell osteogenic and adipogenic differentiation (Elsafadi et al., 2017). TGFβ 2 inhibits C/EBP alpha and C/EBPβ to enhance serine phosphorylation of PPAR gamma, thereby inhibiting adipogenic differentiation of MSCs (Ahdjoudj et al., 2005). Early studies reported that tumor exosomes are closely related to tumor development, but recent studies have also shown that tumor exosomes also play a role in adipogenesis (Wang et al., 2016). As mentioned earlier, exosomes contain miRNAs that can regulate adipogenic differentiation, and studies have shown that adipose tissue mesenchymal stem cells (ADMSCs) can ingest lung tumor cell A549-derived exosomes, which inhibit adipogenic differentiation with decreased PPAR gamma expression. There are many pathways of adipogenesis, and the increase of phosphorylated Smad2 was reversed when the TGFβ inhibitor SB431542 was added (Wang et al., 2017). At the same time, the TGFβ pathway is activated, allowing the absorption of A549-derived exosomes to inhibit adipogenic differentiation (Ahdjoudj et al., 2005). The related miRNA and proteins of A549 cell line activation TGFβ pathway have not been revealed, so its specific mechanism needs further exploration.

The absorption of exosomes secreted by tumor cells usually triggers the inhibitory pathway of adipogenic differentiation. This results in more severe fat tissue defects in cancer patients, which prevent the formation of fat tissue. This means that cancer exosomes can be used as regulators of fat decomposition.

### Erythromyeloblastoid Leukemia Cell→miR-92a-3p→C/EBP α

In previous studies, miR-92a-3p has been found to enhance chondrogenesis by targeting WNT5A protein (Mao et al., 2018). There is a balanced relationship between osteogenesis and adipogenesis. Since miR-92a-3p is associated with osteogenesis, it may also be associated with adipogenesis. Studies reported that miR-92a-3p could regulate adipogenesis (Wan et al., 2019). MiRNA sequencing was performed in exosomes secreted by human erythromyeloblastoid leukemia cell and the characteristics of exosomes were analyzed. It was found that there were a large number of miR-92a-3p in both cells and exosomes (Wan et al., 2019). MiR-92a-3p is transferred into ADSCs by exosomes to participate in cell differentiation, and down-regulates C/EBPα and some lipogenic genes by directly targeting C/EBPα. Low expression of C/EBPα can inhibit adipogenic differentiation of ADSCs (Zhang et al., 2004).

Tumor-associated exosomes uptake by adipocytes inhibited its lipogenesis. Mainly because it contains miRNA that reduces the expression of adipogenic gene C/EBPα, PPAR-γ in adipocytes. This is also consistent with the loss of adipose tissue in cancer patients.

## Potential Pathways of Exosome Regulation of Adipogenesis

As shown above, exosomes contain abundant RNAs and proteins, which play a regulatory role in adipogenesis by being endocytosed by cells. In general, the components contained in exosomes are mainly involved in adipogenic differentiation by affecting the expression of C/EBP and PPAR or signaling pathways affecting adipogenesis. In addition to the aforementioned influence factors, there may be some potential targets.

### Human Umbilical Cord Mesenchymal Stem Cell-Derived Exosomes (HUMSC)→TGFβ

Human umbilical cord mesenchymal stem cell (HUMSC) has been continuously explored due to their non-invasive accessibility, low immunogenicity and high differentiation ability (Wang et al., 2004). Studies have shown that HUMSC can differentiate into adipocytes, chondrocytes, skeletal muscle and endothelial cells under certain induction conditions (Xing et al., 2016; Ji et al., 2020). HUMSC can differentiate into adipocytes, which suggest that the exosomes secreted by HUMSC may be involved in the adipogenic process. The experiment demonstrated that HUMSC secreted exosomes inhibited TGFβ1 signal (Hu et al., 2020). TGFβ1 is not only the key factor of fibroblast-myofibroblast transformation, but also the factor of inhibiting adipogenic differentiation. We speculate that HUMSC secreted exosomes may promote adipogenic differentiation by inhibiting TGFβ1 signal.

### BM-MSC→IGF-1

BM-MSC improves renal insufficiency by local release of insulin-like growth factor 1 (IGF-1). In the exploration of exosomes secreted by human BM-MSC (hBM-MSC), IGF-1 receptor gene was found (Tomasoni et al., 2013). When these receptor genes are absorbed, the release of IGF-1 in BM-MSC can be promoted. Pre-adipocytes have a large number of IGF-1 receptors those bind to released IGF-1 to induce pre-adipocyte differentiation (Rabiee et al., 2018). The full-length pre-adipocyte factor (Pref-1) inhibits pre-adipocyte differentiation, while IGF-1 can skip the blockade of Pref-1 (Zhang et al., 2003). The primary cilium is generated during the arrest phase of pre-adipocyte growth. The presence of primary cilia elevates the binding of IGF-1 to the receptor, prompting pre-adipocytes to continue to differentiate into adipocytes (Zhu et al., 2009). This suggests that BM-MSC may promote lipid differentiation by increasing the level of IGF-1.

### Neuronal Exosomes→Proline-Rich 7 (PRR7)→WNT

Endogenous PRR7 has been demonstrated to exist in exosomes secreted by central neurons (Murata et al., 2005). Overexpression of PRR7 eliminates excitatory synapses in hippocampal nerves and decreases the amount of Wnt7a in exosomes. Removal of PRR7 increases the expression of Wnt5a and Wnt7a secreted from exosomes, which suggests that PRR7 has a role in inhibiting WNTS secretion (Lee et al., 2018). β-catenin is an important regulator of adipogenesis (Karczewska-Kupczewska et al., 2016). WNT binding to the receptor leads to inactivation of the β-catenin destruction complex (Komiya and Habas, 2008). The inactivation of the β-catenin destruction complex prevents the degradation of β-catenin. If β-catenin is not degraded, it can stably activate WNT target genes in the nucleus to activate WNT signaling pathway (Bowers and Lane, 2008). WNT signaling pathway can inhibit the terminal differentiation of pre-adipocytes to achieve the purpose of inhibiting adipogenesis (Davis and Zur Nieden, 2008; Bowers and Lane, 2008). Therefore, PRR7 may promote adipogenesis by inhibiting the WNT signaling pathway.

## Applications of Exosomes in the Adipose Tissue Engineering

Adipose tissue engineering can repair soft tissue defects and correct contour deformations, providing a solution for tissue reconstruction after soft tissue trauma. In recent years, ADSCs have been considered as ideal seed cells for adipose tissue engineering (Karagoz et al., 2019). However, many studies have shown that the reconstruction of adipose tissue produced by adipogenic differentiated MSCs is limited due to the shortage of oxygen and nutrients, so the formation of new blood vessels is very important in constructing adipose tissue engineering (Wang et al., 2018a). Exosomes play an important role in promoting angiogenesis. For example, injection of exosomes secreted by adipose stem cells to the skin flaps can improve flaps repair, protect the flaps, and induce the formation of new blood vessels (Karagoz et al., 2019). Due to exosomes have properties such as promoting cell proliferation and differentiation, and promoting angiogenesis, they have great potential to be used as a cell-free therapeutic approach in adipose tissue engineering (Dai et al., 2017). An engineered bioactive exosomal hydrogel (FHE hydrogel) has been invented to promote neovascularization and accelerate granulation tissue formation at the wound site (Wang C. et al., 2019). Several years ago, it was discovered that combining adipose derived stem cells with decellularized human adipose tissue extracellular matrix could be used for adipose tissue engineering (Wang et al., 2013). Recent studies have found that exosomes secreted by hADSCs can induce adipose tissue regeneration by combining with decellularized adipose tissues scaffold (Nie et al., 2020). In comparison, exosomes are less immunogenic and can be more conveniently injected into adipose tissue engineering scaffolds. Notably exosomes have better specialization in promoting adipogenic direction.

## Discussion

A lot of investigations show exosomes play an important role in determining the fate of stem cell differentiation. The functions of exosome participating in MSCs adipogenic differentiation mainly depends on the RNA and proteins it contains. Different origin of the exosomes may contain different types of contents and play different roles. For example, umbilical cord mesenchymal stem cell-derived exosomes are rich in miR-21, miR-23a, miR-125b, and miR-145, which can suppress myofibroblast differentiation (Fang et al., 2016). HMSC source exosome contains a large amount of miR-92a-3p that can promote chondrogenesis (Mao et al., 2018). miR-18a and miR-182 inclusion in ADSCs-derived exosomes can promote neurite outgrowth (Ching et al., 2018).

In the current researches on exosomes, exosomes mainly indirectly affect adipogenesis, and the regulation mode is simple. The exosomes involved in these studies are almost all influenced by endocytosis, which is dose-dependent, energy-dependent and protein-dependent. The effects of other functions, such as exosome and ECM protein binding, have been rarely studied. Researchers have demonstrated that exosomes can bind to fibronectin and type I collagen (Huang et al., 2016). The extracellular environment is closely related to the proliferation and differentiation of MSCs. The effect of exosomes binding to ECM proteins on cells is worth exploring in further study.

Exosomes play an important role in cellular communication. Due to its non-cellular properties, it is easy to transfer contents, such as mediating endocytosis and acting as a carrier. In the study of exosomes as drug carriers, many applications of delivering targeted drugs were applied to tumors treatment (Wang Y. et al., 2020). In subsequent studies to explore adipogenic differentiation, exosomes can be used as vectors to deliver miRNAs that we want to explore to target cells.

The mechanism of exosomes causing adipose tissue loss in the tumor direction has been intensive exploration. Tumor exosomes can inhibit lipogenesis by triggering signaling pathways, such as TGF and WNT, or inhibiting the expression of lipid-generating genes. Tumor exosomes should be regarded as an important factor in the decomposition of fat, which can be used as a candidate for preventing adipogenesis.

In order to improve the targeting ability, carrying capacity and specificity of exosomes, exosomes can be modified and processed by various improvement strategies. The EV modification could be achieved through direct modification of EVs or modification of the cells of origin used for EV production. Taking advantage of the porous nature of exosome membranes, physical methods such as electroporation and sonication can be used to load drugs and viruses directly to exosomes. Other chemical methods, such as Lipofection and drug incubation, can also directly improve exosome carrying capacity (Xu et al., 2020). Modification of EV originating cells that allows subsequent isolation of EVs, which already express the desired molecule. Tumor necrosis factor (TNF)-related apoptosis-inducing ligand (TRAIL) transduced leukemia cells can produce TRAIL^+^ secreted exosomes (Rivoltini et al., 2016). This can modify the surface of exosomes, improve the receptor binding rate and enhance the targeting ability of exosomes. Further improvement of drug loading strategies and modification methods for engineered exosomes will be appreciated in future studies.

Exosomes not only affect adipogenesis by affecting the expression of lipid-generating genes C/EBP and PPAR, but also indirectly regulate adipogenesis by activating or inhibiting adipogenic signaling pathways. Due to the abundance of exosomes, the potential of exosomes to regulate MSCs differentiation is unlimited.

In adipose tissue regeneration, in addition to the adipogenesis, the lack of vascularization is another issue to overcome. Exosomes as a major form of intercellular communication, play essential roles in angiogenesis. Furthermore, recent studies demonstrate that several factors are involved in regulating the proangiogenic properties of exosomes (Anderson et al., 2016; Xue et al., 2018). The application of exosomes will enhance blood vessel formation which contribute to vascularized adipose tissue regeneration.

The current studies have shown that exosomes have great potential on adipogenic differentiation of mesenchymal stem cells, but the results are collected from the studies from cell and animal. There are no clinical trials on evaluating the exosomes on adipose formation in human. It is very important in clinics on exploring effectiveness and safety of exosomes.

## Conclusion

**FIGURE 2:**
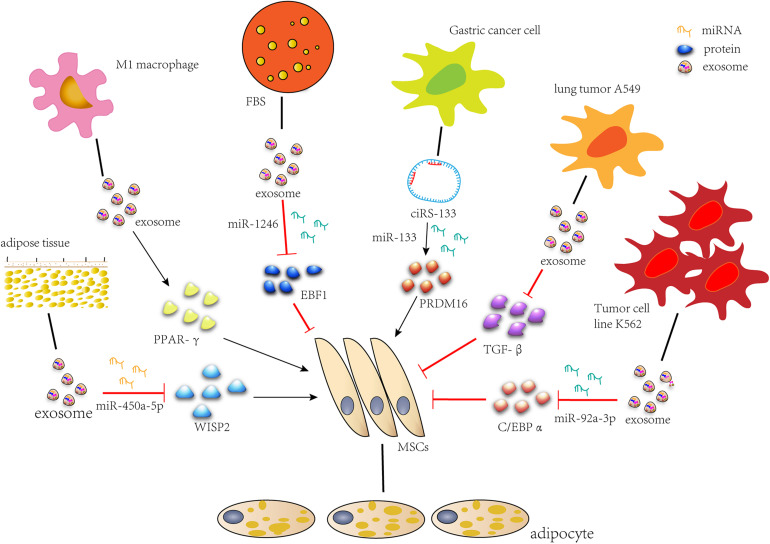
Exosomes regulate adipogenic differentiation. miR-450a-5p contained in exosomes from rat adipose tissue promotes adipogenesis by inhibiting the expression of WISP2. Exosomes secreted by M1 macrophages significantly promote lipid droplet formation by up-regulating the expression of PPAR-gamma gene. miR-1246 in FBS secreted exosomes inhibits adipogenic differentiation by targeting EBF1. Gastric cancer exosome-delivered ciRS-133 promotes adipogenic differentiation by suppressing the functionality of miR-133 and promoting PRDM16 expression. Lung tumor cell A549-derived exosomes inhibit adipogenic differentiation via TGFβ signaling pathway. miR-92a-3p in K562 cell line inhibits adipogenesis of ADSC by reducing C/EBP α expression after transcription.

During recent years, the role of exosomes in fat formation has been developed, and related studies have emerged. We summarized the previous work and formed the molecular network of the regulatory role of exosome in the lipid formation process of MSCs (Figure 2). We conclude that exosomes secreted by MSCs or stem cells associated with fat, as well as M1 macrophages and FBS, promote adipogenic differentiation, while exosomes secreted by tumors inhibit it. When adipogenesis is induced or tissue engineering adipose tissue is constructed, exosomes can be used to deliver RNAs, proteins and small drugs as a carrier. Tumor exosomes can be used when fat decomposes. In addition, an accurate setting of therapeutic doses of exosomes should also be considered. The utilization of exosomes to treat adipose tissue defect has a great potential clinical significance.

## Author Contributions

YZ, XL, FW, and JF: writing and editing. JF: conceptualization. SW: visualization of exosome by TEM. XW, XT, and SB: detailed techniques contribution. DM collected some literature. All authors contributed to the article and approved the submitted version.

## Conflict of Interest

The authors declare that the research was conducted in the absence of any commercial or financial relationships that could be construed as a potential conflict of interest.
